# *GATA4* and *DcR1* methylation in glioblastomas

**DOI:** 10.1186/1746-1596-8-7

**Published:** 2013-01-15

**Authors:** Paulina Vaitkienė, Daina Skiriutė, Kęstutis Skauminas, Arimantas Tamašauskas

**Affiliations:** 1 Laboratory of Neurooncology and Genetics, Neuroscience Institute, Kaunas Academy of Medicine, Lithuanian University of Health Sciences, Eiveniu str. 4, Kaunas, LT, 50009, Lithuania

**Keywords:** Glioblastoma, Methylation, *GATA4*, *DcR1*

## Abstract

**Background:**

Epigenetic silencing of tumor suppressor genes plays important role in gliomagenesis. Recently, *GATA4* and *DcR1* were suggested to be a tumor suppressor genes involved in tumorigenesis in various types of human cancers. However, up to now the methylation frequency of *GATA4* and *DcR1* genes has not been determined in glioblastoma. In this study, we investigated methylation of *GATA4* and *DcR1* promoters and their association with patient prognosis in glioblastoma.

**Methods:**

Methylation status of *GATA4* and *DcR1* promoters was investigated by methylation specific PCR in 99 glioblastoma patients. Statistical analyses were conducted to investigate the association between clinical variables and overall survival time.

**Results:**

*GATA4* and *DcR1* were aberrantly methylated in 23.2% and 27.6% of glioblastoma tumors, but not in normal brain. *GATA4* promoter hypermethylation showed significant association with patients age (p = 0.027). Relationship between genes promoter methylation and glioblastoma patient survival was not determined.

**Conclusions:**

The present work demonstrated that *GATA4* and *DcR1* promoter hypermethylation is tumor specific event in glioblastoma but they promoter methylation cannot be considered as a prognostic marker of glioblastoma survival.

**Virtual Slides:**

The virtual slide(s) for this article can be found here: http://www.diagnosticpathology.diagnomx.eu/vs/1381170351801852

## Background

Glioblastoma multiforme (GBM) is the most common and most aggressive malignant primary brain tumor in humans. Despite multimodality treatment, prognosis is poor, with a median survival time of approximately 14 months. Therefore looking for new markers which would help better diagnose and predict the course of glioblastoma is important. Understanding apoptosis in disease conditions is very important as it not only gives insights into the pathogenesis of a disease but may also leave clues on how the disease can be treated [1]. Apoptosis activation can be triggered either by engagement of cell surface “death receptors” or by cellular stress [2]. The proteins of Bcl-2 family are key regulators of the stress-induced apoptotic pathway [2]. Although, much is known about the pathways and mechanisms of apoptosis, but the significance of some genes is not fully clear. Our interests have focused on two genes *GATA4* and *DcR1*, which are related to apoptosis. Both of these genes are located on chromosome 8p region. Despite 8p being a relatively small chromosome arm, it is one of the most frequently altered genomic regions in human cancer, and is also rich in candidate oncogenes and tumor suppressor genes associated with the development of certain types of cancers [3]. Deletions in this region are observed in glioblastoma [4]. As an alternative to deletion *GATA4* and *DcR1* promoter methylation may be associated with clinical data of glioblastoma patients. *GATA4* is a member of the GATA family of zinc finger transcription factor, which regulates gene transcription by binding to GATA elements. *GATA4* serves as a survival factor in cancer cells by regulating the expression of anti-apoptotic Bcl-2 and Bcl-x L [5]. Although *GATA4* was expressed in normal brain, loss of *GATA4* expression was observed in 57.6% (94/163) GBM operative samples and it was a negative survival prognostic marker [6]. According to these data was formed hypothesis that promoter methylation of *GATA4* gene could be survival prognostic marker.

The protein encoded by *DcR1* gene is a member of the TNF-receptor superfamily. This receptor is not capable of inducing apoptosis, and is thought to function as an antagonistic receptor that protects cells from TRAIL-induced apoptosis. The expression of this gene was detected in many normal tissues but not in most cancer cell lines, which may explain the specific sensitivity of cancer cells to the apoptosis-inducing activity of TRAIL [7]. Methylation of *DcR1* gene promoter was determined in 21% of low grade gliomas [8] suggesting its role in gliomagenesis.

In this study, we evaluated the methylation status of *GATA4* and *DcR1* promoters in glioblastoma tumor tissue to support the hypothesis that they are inactivated in glioblastomas by promoter hypermethylation and plays a role as tumor suppressor genes in gliomagenesis.

## Methods

### Glioblastoma tumor tissue and normal brain

In total, 99 glioblastoma tumor samples from initial surgery were collected in Neurosurgery clinics of Hospital of Lithuanian University of Health Sciences (Kaunas, Lithuania) through 2003 to 2009 yr. Written patient consent under the approval of ethics committee of Lithuanian University of Health Sciences was obtained for every patient. Data base closure was in November 2010. Glioblastoma Multiforme WHO grade IV (Original SNOMED ID M-94403) diagnoses were established by experienced pathologists according to the World Health Organization (WHO) classification. We included Human brain DNA (Zymo Research, USA) as normal brain control. Glioblastoma samples were stored in liquid nitrogen before DNA extraction. The following clinical data were determined for each patient: age at the time of the operation, gender, tumor multifocality, time of the last follow-up and patient status. Survival time was collected for all cases and overall survival calculated from the date of the operation to death, or last contact to the live patients.

### DNA isolation and bisulfate modification

Tumor DNA was extracted from 25-40 mg of frozen tissue using ZR Genomic DNA™ Tissue MiniPrep (Zymo Research, USA) according to manufactures protocol. The methylation status of *GATA4* and *DcR1* promoters was determined by bisulfite treatment of DNA. 400 ng DNA was used for bisulfite modification. DNA modification was performed using EZ DNA Methylation Kit (Zymo Research, USA), and all procedures were done according to manufactures protocol. Bisulfite treated DNA was eluted in 40 μl distilled water, and stored in −80°C until MS-PCR.

### Methylation-specific PCR

The methylation status of the *GATA4* and *DcR1* promoters region was determined by methylation-specific PCR. Primers distinguishing unmethylated (U) and methylated (M) alleles were taken from articles [9,10] are shown as follows in Table 1.

**Table 1:** Primers for MSP

**Gene**	**Forward primer 5**^**′**^**-3**^**′**^	**Reverse primer 5**^**′**^**-3**^**′**^	**Tm,°C**
*GATA4-M*	GTATAGTTTCGTAGTTTGCGTTTAGC	AACTCGCGACTCGAATCCCCG	62
*GATA4-U*	TTTGTATAGTTTTGTAGTTTGTGTTTAGT	CCCAACTCACAACTCAAATCCCCA	62
*DcR1-M*	TTACGCGTACGAATTTAGTTAAC	TCAACGACCGACCGAAACG	60
*DcR1-U*	GAATTTTTTTATGTGTATGAATTTAGTTAAT	CCATCAAACAACCAAAACA	60

M – methylated, U – unmethylated, Tm – melting temperature.

Each PCR reaction contained 20 ng of sodium bisulphite-modified DNA. MSP reaction was performed in 20 μl of total volume, using 10 μl Maxima® Hot Start PCR Master Mix (Thermo fisher Scientific, USA) with Hot start Taq DNA polymerase and 10pmol of each primer (Metabion International AG, Germany). Cycling conditions were initial denaturation at 95°C for 5 min, 38 cycles of 94°C for 30 s, 60/62°C for 1 min and 72°C for 1 min and final step at 72°C for 5 min. For each set of methylation specific PCR reactions human blood lymphocyte DNA treated with bisulfite served as a unmethylated DNA control and as positive methylation control was used ‘Bisulfite Converted Universal Methylated Human DNA Standard’ (Zymo Research, USA). A water blank control was also included. PCR products were separated on 2% agarose gels with ethidium bromide and visualized under UV illumination. PCR analyses were repeated.

### Statistical analysis

SPSS Statistics 19 (SPSS Inc., Chicago, IL) software package was used for statistical analysis. The quantitative data presented as median and to show the reliability of the estimate, the confidence interval (CI) with 95% confidence level was presented. Association between gene methylation data and clinical features of glioblastoma patients were evaluated by using Fisher exact test. To estimate survival functions Kaplan-Meier method was used. For comparing survival time distribution between groups the log-rank test was used. A *p* < 0.05 was considered significant.

## Results

### Characteristics of glioblastoma patients

The median age at diagnosis was 61.0 yr. (CI: 57.3-64.7). The male to female ratio was: 1:1.3. The median age of males (n = 42) was 58.0 yr. (CI: 52.1-63.9) and females (n = 57) 61.0 yr. (CI: 56.9-65.1). Median survival time of glioblastoma patients (n = 99) was 8.9 months (CI: 6.9-10.8). Most of glioblastoma patients (64%) survived less than 12 months after operation.

### *GATA4* and *DcR1* promoters are methylated in glioblastoma

**Figure 1:**
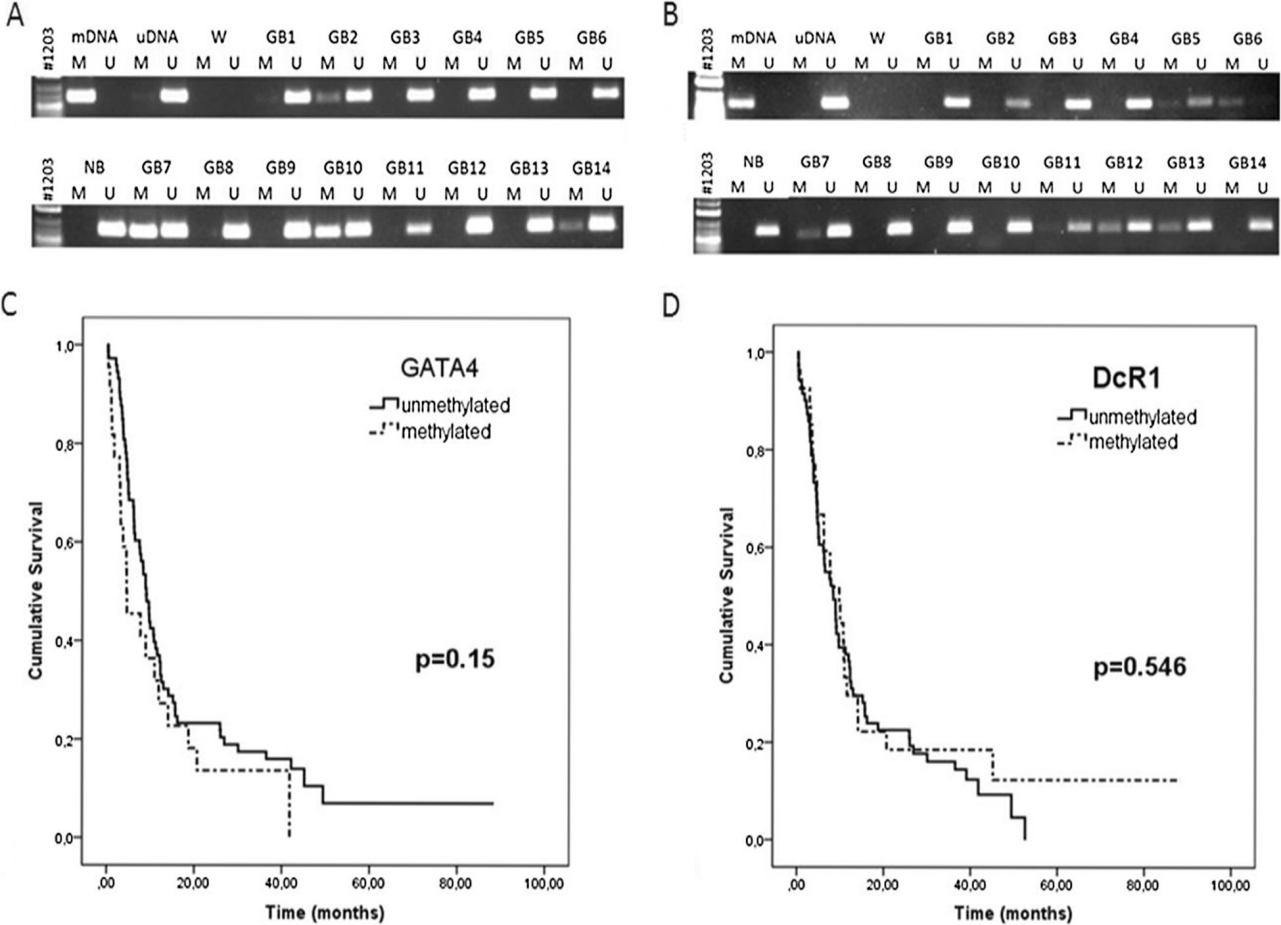
**Analysis of *GATA4 *and *DcR1 *in glioblastoma; Analysis of *GATA4 *(A) and *DcR1 *(B) CpG island promoter methylation status in glioblastoma by the methylation-specific PCR assay.** Molecular weight markers are shown in the left, mDNA- methylated DNA control, uDNA-unmethylated DNA control, W- water control, GB1-GB14 glioblastoma tumor samples, NB normal brain sample. The presence of visible PCR products in those lanes marked “U” indicates the presence of unmethylated allele genes and “M” indicates the presence of methylated genes. Kaplan-Meier cumulative survival (months) in glioblastoma patients according to methylation status of (**C**) *GATA4* (log-rank test, *p* = 0.15) and (**D**) *DcR1* (log-rank test, *p* = 0.546).

The methylation status of the *GATA4* and *DcR1* promoter in glioblastoma samples was detected by methylation-specific PCR assay. We evaluated methylation status of the *GATA4* promoter in 95 glioblastoma tumors. Promoter hypermethylation was detected in 23.2% (22/95) of the glioblastoma but not in normal brain. Representative samples are shown in Figure 1. The same studies were performed with *DcR1* gene promoter in 98 glioblastoma samples. *DcR1* gene promoter hypermethylation was detected in 27.6% (27/98) of the glioblastoma tumors but not in normal brain.

The detection of bands with both primer sets were found in some but not all of the glioblastoma samples likely because of the existence of non-malignant cells in a fraction of the samples or that only one allele of the gene is methylated.

Among 22 *GATA4*-methylated cases, five patients (23%) were younger than 60 years and 17 individuals (77%) were older than 60 years. There is significant difference in gene methylation ratio between age groups (Fisher’s Exact test 2-sided, *p* = 0.027; Table 2) and this means that *GATA4* methylation is related to age. Meanwhile, the interface between the *DcR1* promoter methylation status and age is unspecified. No dependences were found between *GATA4* and *DcR1* promoter methylation and two-year survival time. These genes promoter methylation was not significantly associated with gender or tumor multifocality too (Table 2). In no one of multifocal glioblastomas *DcR1* promoter methylation was observed. Maybe the study of a large number of samples could show that *DcR1* is an important marker. We analyzed *GATA4* and *DcR1* promoter methylation for prognostic value of overall survival (OS) using Kaplan-Meier Curves (*p* values were generated using the log-rank test) (Figure 1C, D). Analysis showed no association between *GATA4* and *DcR1* gene promoter methylation and survival in glioblastomas.

**Table 2:** ***GATA4 *and *DcR1 *promoter methylation in human glioblastoma tissue with different clinicopathological features**

**Variables**	**GATA4**		**Statistic significance**	**DcR1**		**Statistic significance**
	**Methylated**	**Unmethylated**		**Methylated**	**Unmethylated**	
**Overall**	22	73		27	71	
**Age (years)**			0.027			1.000
<60 years	5	37		12	32	
≥60 years	17	36		15	39	
**Gender**			0.473			0.263
Male	11	30		9	33	
Female	11	43		18	38	
**Multifocal**			1.000			0.185
Yes	2	5		0	7	
No	20	68		27	64	
**Survival (months)**			0.227			1.000
<24	20	57		22	57	
≥24	2	16		5	14	
***DcR1***			0.185			-
Methylated	9	18		-	-	
Unmethylated	13	53				

## Discussion

Understanding of the molecular alterations that occur during tumorigenesis, and identification of novel markers for cancer diagnosis and novel targets for treatment, may be important for the improvements in tumor diagnosis, treatment and prevention [11]. The most common molecular alterations, that characterize glioblastomas and could be used in current clinical practice and therapeutic decision making, are proliferation markers (Ki-67/MIB-1 and phospho-histone-H3 (PHH3)), mutations involving isocitrate dehydrogenase (IDH1 and IDH2) and *TP53*, also 1p/19q deletion, mutations of Epidermal growth factor receptor (EGFR), O-6-methylguanine-DNA methyltransferase (*MGMT*) promoter methylation status and glioma-CpG island methylator phenotype [12-14]. Despite extensive efforts at defining biological markers, therapeutic progress for glioblastoma is slow. A further understanding of glioma biology, in concert with well-designed clinical trials, is necessary to identify more putative molecular biomarkers and unravel the mysteries in the pathogenic mechanisms that trigger this menacing disease [12]. The identification of methylated genes in cancer may provide an insight in the molecular mechanisms of tumor development and might reveal new tools to define markers of prognostic significance [15]. In order to identify molecular markers relevant to glioblastoma development, diagnosis and prognosis promoter methylation analysis of 2 genes, *DcR1* and *GATA4*, was performed.

Loss of *GATA4* expression due to promoter hypermethylation has been reported in primary colorectal, gastric, esophageal, lung and ovarian cancer [10,16]. To analyze the potential of *GATA4* as methylation marker in glioblastoma, we analyzed large series of glioblastoma tumor samples and one normal brain tissue. Were showed that methylation of *GATA4* (23.2%) occurs not at so high frequencies in glioblastoma as in colorectal cancer (70%) [10] or sporadic gastric carcinomas (53.8%) [10]. Loss of *GATA4* was observed in about 57.7% GBM operative samples [6]. In our study *GATA4* methylation was detected only in 23.2% of cases, and this may suggest that the *GATA4* expression is regulated by different mechanisms or mutation. This could explain the lack of links between GATA4 promoter methylation and survival (p = 0.15) in our study contrary to results obtained in Agnihotri et al. (2011) expression studies [6].

A tumor necrosis factor receptor or death receptor family members were found frequently methylated in glioblastoma: TNFRSF10D promoter methylated in 100% [8], and TNFRSF10A in 68% [11] of glioblastomas. Methylation of *DcR1* gene promoter was determined in 21% of low grade gliomas (LGGs; World Health Organization [WHO] grade II) [8]. There is not much data about *DcR1* promoter methylation in glioblastoma. Martinez et al. (2007) in 16 glioblastoma patients did not found methylation of *DcR1* promoter [17]. We have assumed that in glioblastomas this percentage should be much higher. Our research has established that methylation of *DcR1* was in 27.6% of glioblastoma samples, but not in the normal brain DNA, which implied that epigenetic silencing of the *DcR1* might be involved in gliomagenesis. This number is close to 37% in lung cancer cell lines or 30.7% in ovarian tumors and is very different from 78.0% methylation in prostate cancer [18-20]. These findings suggest that the prognostic value of promoter hypermethylation of *DcR1* could be tissue-specific. The study of gene methylation impact on survival found that the methylation is not associated with survival (p = 0.54). Although promoter hypermethylation of *GATA4* and *DcR1* genes were found in glioblastoma, they were not associated with patient sex or tumor multifocality in our series. Meanwhile, it was found that GATA4 methylation is related to age (p = 0.027).

## Conclusion

In conclusion, the present work demonstrated that *GATA4* and *DcR1* promoter hypermethylation is tumor specific event in glioblastoma but they promoter methylation cannot be considered as a prognostic marker in glioblastoma.

## Competing interests

The authors declare that they have no competing interests.

## Authors’ contributions

PV did all gene methylation analysis, generated the main idea of the article, did all statistical analysis and wrote the manuscript. DS did DNA extraction and DNA bisulfite modification and contributed to statistical analysis. KS and AT gathered patient tumor sample and clinical data, supervised the project. All authors read and approved the article.
